# Combined genetic influence of the nicotinic receptor gene cluster *CHRNA5/A3/B4* on nicotine dependence

**DOI:** 10.1186/s12864-018-5219-3

**Published:** 2018-11-20

**Authors:** Sung-Ha Lee, Woo-Young Ahn, Michał Seweryn, Wolfgang Sadee

**Affiliations:** 1 Center for Pharmacogenomics, Department of Cancer Biology and Genetics, College of Medicine, The Ohio State University, 1004 Biomedical Research Tower, 460 W 12th Avenue, Columbus, OH USA; 2 Department of Psychology, The Ohio State University, 1835 Neil Avenue, Columbus, OH USA; 3 Center for Medical Genomics OMICRON, Jagiellonian University, Medical College, Krakow, Poland; 4 Center for Happiness Studies, Seoul National University, Gwanak-gu, Gwanak-ro 1, Bldg. 220, Seoul, 151-746 South Korea; 5 Department of Psychology, Seoul National University, Gwanak-gu, Gwanak-ro 1, Bldg. 16, Seoul, 151-746 South Korea

**Keywords:** *CHRNA5*, *CHRNA3*, *CHNRB4*, rs16969968, rs880395, rs1948, rs4887074, eQTL, Nicotine dependence, Haplotype, Diplotype, Odd ratios, Area under the curve, Heteroscedasticity

## Abstract

**Background:**

The *CHRNA5/A3/B4* gene locus is associated with nicotine dependence and other smoking related disorders. While the non-synonymous *CHRNA5* variant rs16969968 appears to be the main risk factor, linkage disequilibrium (LD) bins in the gene cluster carry frequent variants that regulate expression. Pairwise LD and haplotype analyses had identified at least three haplotype tagging SNPs including rs16969968 as main genetic risk factors. Searching for variants with evidence of regulatory functions, we have reported interactions between *CHRNA5* and *CHRNA3* enhancer variants (tagged by rs880395 and rs1948, respectively) and rs16969968, forming 3-SNP haplotypes and diplotypes that may more accurately reflect the cluster’s combined effects on nicotine dependence (Barrie et al., Hum Mutat 38:112–9, 2017). Here we address further contributions by variants affecting *CHRNB4*, a possibly limiting component of nicotinic receptors.

**Results:**

We identify an LD bin (tagged by rs4887074) associated with expression of *CHRNB4*. Additive logistic regression models indicate that rs4887074 is associated with nicotine dependence and modulates the effect of rs16969968 in GWAS datasets (COGEND, UW-TTURC, SAGE). 4-SNP haplotype and diplotype analyses (rs880395-rs16969968-rs1948 -rs4887074) yield nicotine dependence risk values that further differentiate those obtained with the 3-SNP model. Moreover, both the main *G* allele of rs16969968 and the minor *G* allele of rs4887074 (associated with reduced expression of *CHRNB4*), residing predominantly on common haplotypes that are protective, represent significant allele-specific variance QTLs, indicating that they interact with each other.

**Conclusions:**

These results indicate rs4887074 is associated with *CHRNB4* expression, and along with two regulatory variants of *CHRNA3* and *CHRNA5*, modulates the effect of rs16969968 on nicotine dependence risk. Assignable to individuals because of strong LD structures, 4-SNP haplotypes and diplotypes serve to assess the combined genetic influence of this multi-gene cluster on complex traits, accounting for complex LD relationships and tissue-specific genetic effects (*CHRNA5/3*) relevant to the traits analyzed. The 4-SNP haplotypes account at least in part for previous tagging SNPs, including the highly GWAS-significant rs6495308, located in a distinct pair-wise LD bin but included in protective 4-SNP haplotypes. Our approach refines and integrates the cluster’s overall genetic influence, an important variable when integrating the genetics of multiple genomic loci.

**Electronic supplementary material:**

The online version of this article (10.1186/s12864-018-5219-3) contains supplementary material, which is available to authorized users.

## Background

The *CHRNA5-CHRNA3-CHRNB4* gene cluster on chromosome 15 encodes the α5, α3 and β4 subunits of nicotinic acetylcholine receptors (nAChR). Genetic variants in this cluster, in particular the non-synonymous (ns) SNP of *CHRNA5*, rs16969968, yields the strongest genome-wide association with nicotine dependence and smoking related disease such as lung cancer [1–4]. A hallmark of evolutionary selections, long haplotype structures with high frequency in this cluster create a local regulome with multiple genetic variants defining the overall genetic influence of the entire cluster [5]. Previous studies have addressed the genomic architecture and linkage disequilibrium (LD) structure of this gene cluster, showing that the *CHNRA5* nsSNP rs16969968 represents the predominant genetic factor, while other candidate variants may modulate this effect by regulating gene expression [2, 6–9]. In a large GWAS meta-analysis, Liu et al. have identified at least three LD-bin tagging SNPs, including rs16969968, a SNP in the promoter region of *CHRNA5* (rs588765), and a SNP located in the *CHRNA3* locus (rs6495308), all significantly associated with smoking behavior [10]. Derived 3-SNP haplotypes then served to estimate the combined genetic influence of the gene locus [10]; however, the presence of additional LD bins suggested as yet undetected multi-SNP haplotypes.

Taking advantage of emerging genomics databases, such as GTEx (Genotype-Tissue Expression), ENCODE (Encyclopedia of DNA element), and dbGaP (the database of Genotype and Phenotypes), we had identified two regulatory LD bins, represented by a lead candidate variant for *CHRNA3* and *CHRNA5* (tagged by rs1948 and rs880395, respectively, the latter in high LD with rs588765 in Europeans), that modulate influence on nicotine dependence [11]. These two SNPs are either the regulatory variants themselves or in high LD with causative variants, an important criterion when utilizing marker variants, because even small deviations from perfect LD with the functional polymorphism lead to failure in detecting dynamic interactions between gene loci and other factors [12]. Both variants affect regulatory domains that direct the expression of more than one gene each [11], via chromatin looping [13]. 3-SNP haplotypes constructed with rs1948, rs880395 and rs16969968 further refine the association with nicotine dependence when compared to that with rs16969968 alone [9, 11]. These associations are likely trait and tissue specific as rs1948 serves as a regulatory variant for *CHRNA3* only in the basal ganglia but not other tissues, where both *CHRNA3* and *CHRNA5* mRNA expression is associated with rs880395 [11]. These results support the notion that associations with complex clinical traits require an understanding of the combined genetic influence of the entire gene cluster, with distinct regulatory processes between tissues affecting different traits. However, eQTL and LD analyses suggested that additional regulatory variants exist in the *CHRNA5-CHRNA3-CHRNB4* cluster, specifically affecting *CHRNB4* expression. The goal of the present study is to identify these additional variants and reconcile the results with previous GWAS findings on nicotine dependence.

Several eQTLs (from GTEx) affect *CHRNB4* mRNA expression in peripheral tissues but are not detectable in brain because of low expression. Here, we characterize an LD bin with potential *CHRNB4* regulatory variants based on expression in peripheral tissues. We then evaluate the combined influence of all four SNPs haplotypes/diplotypes (rs16969968 and three regulatory variants) on nicotine dependence in GWAS, extending our previous 3-SNP analysis [11]. Because of the extensive LD block structure of the gene cluster, 4-SNP haplotypes and diplotypes are assignable with confidence to a large portion of subjects in a GWAS of nicotine dependence (COGEND, UW-TTURC, SAGE). The results of the 4-SNP haplotype/diplotype analysis confirm and extend previous findings on the genetic influence of the *CHRNA5-CHRNA3-CHRNB4* gene cluster on nicotine dependence and reconcile previous GWAS data. We further use a machine learning approach for the identification of generalizable genetic markers associated with behavioral phenotypes [14, 15]. Unlike a univariate approach comparing groups on each measure separately, this machine learning approach seeks to identify multivariate patterns of all the measures that can predict or classify a phenotype in new samples [16].

Lastly, we ask whether the four key haplotype SNPs show evidence for dynamic interactions among each other or with additional factors, evidenced as differences in the allele-specific variance of phenotype associations, rather than in the mean main effect. Dynamic interactions between variants (epistasis) might well account for part of the ‘missing heritability’ [17]. Significant difference in variance of phenotypic traits between alleles is characterized as a variance QTL (vQTL) [18, 19], revealing dynamic interactions between candidate variants and other factors that remain hidden in GWAS analysis with assumption of linear additive effects. To assess dynamic conditional effects, we apply a technique based on neural networks together with the Breusch-Pagan test for heteroscedasticity to detect vQTLs [20].

## Methods

### Pre-selection of candidate variants across the *CHRNA5-CHRNA3-CHRNB4* gene cluster

To identify putative causal variants, we followed the criteria outlined previously [11] to select variants for *CHRNA5/A3/B4*:

1. Clinical associations/gene expression (GWAS/eQTL hits): To select genetic variants associated with clinical phenotypes, SNPs derived from NHGRI-EBI GWAS Catalog (https://www.ebi.ac.uk/gwas/) and Genome-Wide Repository of Associations Between SNPs and Phenotypes (GRASP) (http://grasp.nhlbi.nih.gov/Overview.aspx) were preselected with *p* < 10^− 5^ and *p* < 0.05 for GWAS and GRASP, respectively. Among these SNPs, we selected genetic variants overlapping with tissue specific expression variants identified as eQTLs. Tissue specific eQTLs were calculated in GTEx using normalized RPKM (Reads Per Kilobase of transcript per Million mapped reads), and imputed genotypes with PEER factors and gender as covariates.
2. Regulatory regions: SNPs in perfect LD (R^2^ = 1, D′ = 1) with GWAS/eQTL hits that reside in regulatory regions defined by ENSEMBL (ENSRs) using BiomaRt R package [21].
3. Gene expression: To select SNP most highly associated with gene expression, eQTLs with the lowest *p* values for *CHRNA5, CHRNA3* and *CHRNB4* in any given tissue in GTEx were selected.
  Using 1000 genomes project data, R^2^ and D′ of LD were calculated using the ‘ld’ function in the ‘snpStats’ R package [22] and based on the R^2^ values, we generate a heatmap that highlight LD bins of candidate SNPs.

### Associations of genotype, haplotypes, and diplotypes with nicotine dependence

To test the association of candidate variants with nicotine dependence, genotypes and clinical phenotype information of Caucasian subjects (*n* = 4410, mean age = 39.5 (± 9.8) years) were obtained from “The study of Addiction: Genetics and Environment (SAGE) (phs000092.v1.p1)” and “The Genetic Architecture of Smoking and Smoking Cessation (phs000404.v1.p1)” which includes COGEND (Collaborative Genetic Study of Nicotine Dependence) and UW-TTURC (University of Wisconsin Transdisciplinary Tobacco Use Research Center) subjects. We used Fagerström Test for Nicotine Dependence (FTND) score (0–10) to measure smoking behavior. Among the participants, subjects whose FTND score is more than 4 were categorized as ‘nicotine dependence’ (*n* = 2821) whereas those whose FTND score was zero and smoked at least 100 lifetime cigarettes were controls (*n* = 840) according to established criteria [23, 24].

#### Logistic regression

Additive logistic regression models were used to evaluate the association between each genetic variant and nicotine dependence. In single variant analysis, a SNP, age and sex were used as independent variables, whereas in the “pairwise snp” analysis: age, sex, rs4887074 and one of the three remaining SNPs (3-SNP haplotype in [11]) were used as predictors. We estimated the effect sizes (beta coefficients) and *p*-values (reported for the standard Wald’s test) using the ‘glm’ function in R.

#### Haplotype/diplotype analysis

Haplotype and diplotype probabilities were estimated via the Expectation Maximization (EM) algorithm as implemented in the Haplo.stat R package [25];. In short, posterior probabilities of all possible haplotypes for each subject were computed and the most significant haplotype and diplotype were selected for further analysis. Individuals with rare haplotype/diplotype (frequency below 2%) were excluded from further analysis. Odds ratios and confidence intervals were calculated from the effect sizes and standard errors. In the estimation step GGGG haplotype or AGAC-GGGG diplotype (most frequent) were set as the base for contrast calculation. To compare the effects between each pair of diplotypes we used the Tukey contrasts as implemented in the multcomp R package [26].

#### Multivariate regression: Machine-learning approach

We evaluated the discriminative power of the previous 3-SNP model compared to a 4-SNP model including rs4887074, with sex and age as co-variants, to test the predictive power of each model with respect to nicotine dependence. For this, we applied the machine learning algorithm Least Absolute Shrinkage and Selection Operator (LASSO) [27]. For fitting LASSO and generating out-of-sample predictions, we used procedures validated previously [14, 15]. In short, we divided the entire dataset into a training (67% of the dataset) and a test set, and estimated the LASSO model using 10-fold cross validation only on the training set, including classification accuracy. Then we obtain out-of-sample predictions on the test set. The process was repeated 1000 times to test model robustness across different training/test divisions. As index of classification accuracy, we determined the area under the curve (AUC) of the receiver operating characteristic (ROC) curve of rs16969968 vs. rs880395/ rs16969968/ rs1948 vs. rs880395/ rs16969968/ rs1948/ rs4887074 using *easyml* R package [28], facilitating use of glmnet R package [29].

#### Search for variance QTL applying heteroscedasticity

To assess the potential of interactions between each of the four SNPs and genetic and/or environmental factors not taken into account in this study, we utilized the concept of heteroscedasticity – i.e., we tested whether the residuals in the proposed linear models are dependent on the SNP. For this approach, we applied the FTND score scale for 0 (non-dependence) or 4 to 10 (nicotine dependence) and tested whether residual probabilities (after regressing out the effect of age and sex) in the logistic regression depend on the genotype of each SNP, to evaluate the influence of the genetic variant on the variability of the phenotype (i.e. FTND score). To this aim we use the Breusch-Pagan test as implemented in the package lmtest in R [30]. The neural network approach focuses on modeling the probability of an individual falling into a whole spectrum of intermediate categories as defined by the nicotine dependence score. Subsequently, we use the residual probabilities (from a model with sex and age as independent variables).

## Results

### Candidate variants across the *CHRNA5-CHRNA3-CHRNB4* gene cluster identified as eQTLs or GWAS hits

**Fig. 1:**
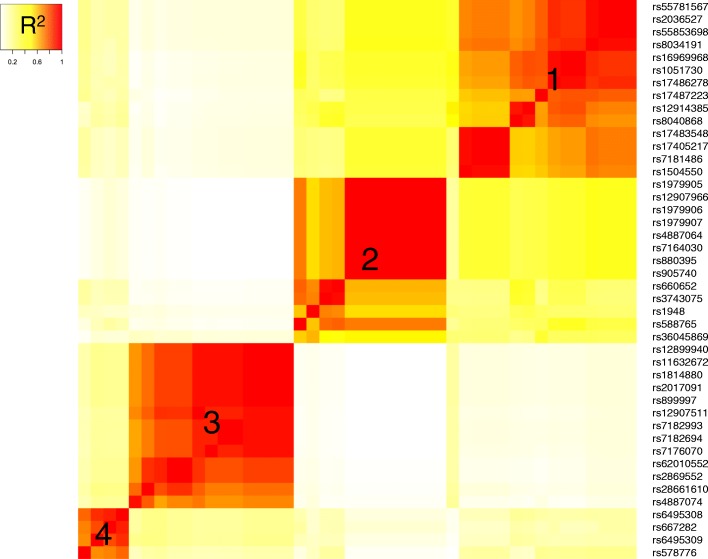
Heatmap of linkage disequilibrium (LD) values (R^2^) between candidate SNPs in the *CHRNA5/A3/B4* locus. Pairwise LD values (Caucasians) were calculated between candidate variants selected by GWAS hits/eQTLs overlap, top eQTLs, and regulatory regions in the CHRNA5/A3/B4 nicotinic receptor locus. On the basis of pairwise LD (R^2^), the candidate SNPs were grouped into four distinct LD blocks; cluster 1 to 4. The heatmap is independent of genomic location of each SNP

The broad search of genomics data on the gene cluster identified 44 candidate SNPs showing evidence for potential function (eQTLs, GWAS hits) that coalesce into 4 groups representing distinct LD bins (Fig 1). For each LD bin, we list the highest scoring SNPs (GWAS or eQTLs) in Table 1, representing lead SNPs. LD bin 1 includes the nicotine dependence risk nsSNP rs16969968 [7, 9, 11, 31], which alters *CHRNA5* receptor function, and variants in high LD with it. LD bin 2 includes the two eQTLs, rs880395 and rs1948 previously identified [11], which increase *CHRNA5/A3* expression in brain and peripheral tissues (rs880395), and *CHRNA3* exclusively in the basal ganglia (rs1948) [11]. LD bin 3 includes variants associated with reduced *CHRNB4* expression, detectable in GTEx only in peripheral tissues (testis and esophagus mucosa), likely because of low expression in the brain tissues. Variants in LD bin 4 are less well characterized by eQTLs but it contains rs6495308, which is strongly associated with smoking quantity in a meta-analysis [10]. Minor alleles of SNPs in bin 4 rarely occur together with the minor alleles of lead SNPs in bin 1 or 2, so that the minor allele of rs6495308 (*C*) resides mostly on the main *G* allele of rs16969968 and rs1948 (Additional file 1: Table S1) while it has lower frequency than rs16969968 (0.24 versus 0.37 in Europeans).

**Table 1:** Characteristics of candidate SNP representing LD blocks in the *CHRNA5/A3/B4* locus

Group	Candidate lead SNP	Asociation with RNA expression^a^	Clinical phenotype^b^
1	rs16969968	nsSNP alters *CHRNA5* receptor function (*CHRNA5*)	Smoking-related, cardiovascular, pulmonary/lung cancer
2–1	rs880395	enhanced expression of *CHRNA5/3* in brain and peripheral tissues	Smoking-related, cardiovascular, pulmonary/lung cancer
2–2	rs1948	enhanced expression of *CHRNA3* in brain basal ganglia	
3	rs4887074	reduced expression of *CHRNB4* in peripheral tissues^c^	Smoking-related Cardio-vascular
4	rs6495308	reduced expression of *CHRNA5/3* in peripheral tissues	Smoking-related, cardiovascular, pulmonary/lung cancer

^a^Based on GTEx

^b^Based on GWAS catalog and dbGaP database

^c^No brain expression eQTLs available in brain regions because of low mRNA expression

Focusing on key candidate regulatory variants of *CHRNA5/A3/B4*, we select representative SNPs by choosing the top-scoring eQTLs (bins 2 and 3) and the nsSNP rs16969968 for LD bin 1. We do not include LD bin 4 variants in haplotype analyses since rs6495308 (bin 4) appears to register as an eQTL because of opposite LD with rs16969968 and rs1948 (rs64953078 is discussed further below). Moreover, rs6495308 is an eQTL for *CHRNA3* in multiple tissues, with *p* = 0.0001 and Normalized Effect Size (NES) = − 0.55 in nucleus accumbens, in nearly the same location as rs2869546, with eQTL values of *p* = 1.7e-13 and NES = − 0.72, whereas rs1948, included in our 4-SNP haplotype, scores with *p* = 6e-15 and NES = − 0.81. These data and LD data (Table 2) question a functional role for rs6495308.

**Table 2:** Pairwise LD of the lead SNPs in European population from 1000 genomes project

	rs880395	rs16969968	rs1948	rs4887074	rs6495308
rs880395	1	0.98 (0.33)	0.83 (0.54)	0.44 (0.04)	0.77 (0.11)
rs16969968		1	0.99 (0.27)	0.41 (0.03)	1 (0.18)
rs1948			1	0.52 (0.04)	0.92 (0.13)
rs4887074				1	0.42 (0.18)
rs6495308					1

D’(R^2^); rs880395 (*G > A*, CHRNA5 enhancer, MAF = 0.38), rs16969968 (*G > A*, *CHRNA5* nsSNP, MAF = 0.37), rs1948 (*G > A*, *CHRNA3* enhancer, MAF = 0.32) and rs4887074 (*C > G*, candidate *CHRNB4* repressor, MAF = 0.24) and rs6495308 (*T* > *C*, MAF =0.24)

*MAF* Minor Allele Frequency

**Fig. 2:**
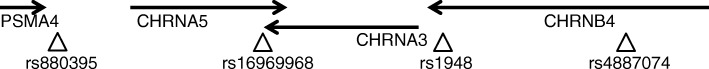
Location of the three regulatory variants and a non-synonymous variant. rs880395 (chr15: 78552014, upstream of *CHRNA5*); rs16969968 (chr15: 78590583, nonsynonymous variant of *CHRNA5*); rs1948 (CHR15: 78625057, synonymous or 3 prime UTR variant located in *CHRNB4*; enhancer for *CHRNA5*) from our previous study, and rs4887074 (chr15: 78659768, intronic variant of *CHRNB4* newly added in the analysis

The selected three regulatory candidate SNPs (rs880395, rs1948, and rs4887074) are located upstream of target genes (Fig 2) and have varying degrees of LD with the ns-SNP rs16969968 (Table 2, which includes rs6495308 for comparative analysis). While R^2^ is rather low (0.03–0.04) between rs4887074 and the other three SNPs, in part accounted for by lower MAF of rs4887074, D’ values are higher (0.41–0.52) (Table 2). Owing to the substantial D’ values across the gene cluster, it is feasible to construct haplotypes and diplotypes of the four SNPs for individual subjects with high confidence.

#### Four candidate SNPs and nicotine dependence

We have previously focused on the *CHRNA3* and *CHRNA5* enhancers (tagged by rs1948 and rs880395, respectively) combined with rs16969968 [11] in European populations from SAGE (Study of Addiction: Genetics and Environment) and the Genetic Architecture of Smoking and Smoking Cessation. Adding here a candidate regulatory *CHRNB4* variant (rs4887074), we use a regression model to estimate haplotypes and diplotypes for the four variants.

### Regression model

Testing the association between each SNP and nicotine dependence in the logistic regression model confirmed previous results that rs16969968 is an individual risk factor for nicotine dependence (effect size = 0.27, *p* < .0001, Additional file 1: Table S2) [11]. In contrast, the minor allele (*G*) of rs4887074, associated with reduced expression of *CHRNB4*, is associated with significant protective effect (negative effect size) in both single SNP and pairwise analysis with other SNPs (Table 3). Although rs880395 and rs1948 are not individually significant (effect size = − 0.09 and − 0.02, *p* = 0.11 and 0.76, respectively) both variants show protective effect when considered in context with rs4887074 (Table 3). This result illustrates the hidden influence of these regulatory variants that interact among each other, confounding single SNP analysis.

**Table 3:** Effect size in nicotine dependence (SAGE) of rs4887074 alone and with rs880395, rs16969968, or rs1948, obtained from a Generalized Linear Model

	rs880395	rs16969968	rs1948	rs4887074
ND~rs4887074				−0.21^***^
ND~rs880395 + rs4887074	−0.14^*^			−0.25 ^***^
ND~rs16969968 + rs4887074		0.24^***^		−0.16^*^
ND~rs1948 + rs4887074			−0.07	−0.23^***^

^*^p < .05, ^**^p < .01, ^***^p < .001 in GLM model

### Associations of genotype, haplotypes, and diplotypes with nicotine dependence

#### Estimation of haplotypes and diplotypes

To evaluate the combined effect of the four candidate variants of the *CHRNA5/A3/B4* locus on nicotine dependence, we build haplotypes and diplotypes and examine the association of the estimated genotype with nicotine dependence. Because of extensive LD structure between rs16969968 (*G > A*), rs880395 (*G > A*) and rs1948 (*G > A*), the minor *A* allele of rs16969968 occurs mainly together with the major alleles of rs880395 and rs1948 (low expression of *CHRNA5/3*). As a result, *GAG* (rs880395-rs16969968-rs1948; major-minor-major allele) and *AGA* (minor-major-minor) are the most frequent haplotypes (35 and 32%, respectively). Adding rs4887074 (*C > G*; the *G* allele being associated with reduced expression), the *GAG* haplotype is divided into *GAGG* (rs880395-rs16969968-rs1948-rs4887074, 5%) and *GAGC* (30%), whereas the *AGA* haplotype is divided into *AGAG* (2%) and *AGAC* (29%). This result demonstrates that the minor *A* risk allele of rs16969968 is predominantly associated with the major alleles of the other three SNPs, including the *C* allele of rs4887074 (further compounding risk compared to the minor rs4887074 *G* allele), and vice versa*.* Moreover, rs4887074 *C > G* splits the next most frequent haplotype, *GGG* (rs880395-rs16969968-rs1948) into *GGGC* (7%) and *GGGG* (13%). The haplotype with all four minor alleles (*AAAG*) is estimated not to occur in this dataset.

**Fig. 3:**
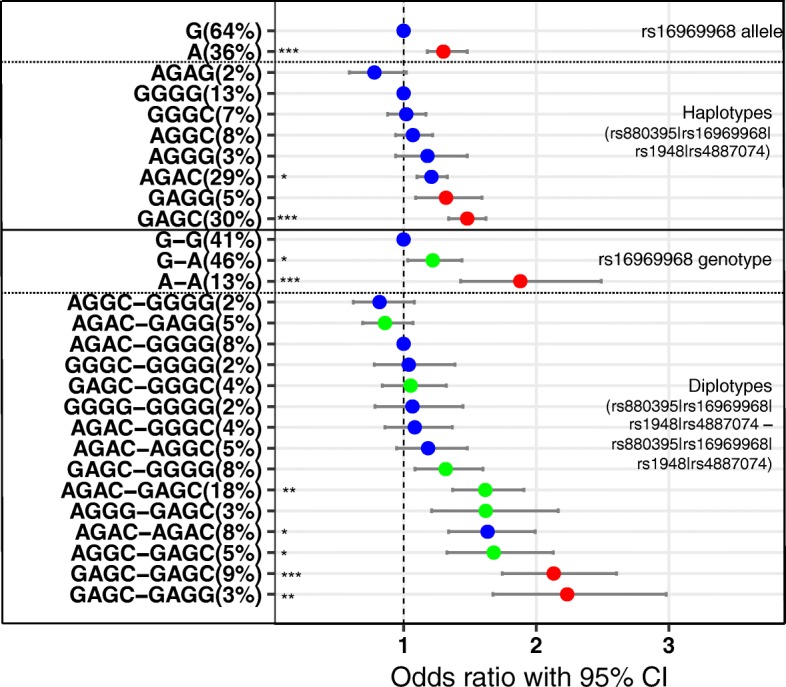
Association of nicotine dependence with rs16969968 allele alone (*G > A*) and haplotype and diplotype comprised of rs880395 (*G > A*), rs16969968 (*G > A*), rs1948 (*G > A*) and rs4887074 (*C > G*), reported as odds ratio with 95% confidence intervals. For haplotype analysis, base allele of rs16969968 and haplotype is G and GGGG respectively. For diplotype analysis, bases are G-G of rs16969968 and AGAC-GGGG for diplotypes. Color represents different allele (or diplotypes) of rs16969968: blue; G (haplotype) or G-G (diplotype), green; G-A and red; A (haplotype) or A-A (diplotype) (*** *p* < .0001, **p < .01,*p < .05, adjusted *p*-value for multiple corrections). The number next to the genotype/ haplotype/ diplotype represents the frequency (%)

Because of the LD structure between four biallelic variants, the number of diplotypes is reduced to 45 assignable diplotypes (Additional file 1: Tables S1 and S3) in the dataset. The posterior probabilities of the haplotypes assigned to each subject range from 0.62 to 1 (median = 0.997, Q1 = 0.932, Q3 = 1) (Additional file 1: Figure S1). Among the diplotypes, we select 15 diplotypes with > 2% frequency, accounting for 86% of the study population, and calculate the odd ratios (OR) and confidence intervals of each diplotype. The most frequent diplotype is *AGAC-GAGC* (18%), the combination of the two most frequent haplotypes. Among the 15 diplotype groups, two diplotypes (red circles in Fig. 3) are homozygous for the minor allele of rs16969968 (*A/A*), six are hererozygous *AG* (green circles), and seven are homozygous *G/G* (blue circles). This haplotype distribution illustrates the skewed nature of haplotypes and diplotypes ranging across the gene locus in long LD blocks.

#### Association of haplotype/diplotype with nicotine dependence in the dataset

Association analysis of the four candidate variants confirms that the *A* allele of rs16969968 is a risk factor for nicotine dependence (Fig. 3, Additional file 1: Table S4). In the haplotype association analysis, the *C* and *G* alleles of *CHRNB4* SNP rs4887074 do not significantly modulate the effect of *GAGC* (rs880395-rs16969968-rs1948-rs4887074) compared to *GAGG* (Fig 3). In contrast, among haplotypes with the main *G* allele of rs16969968, rs4887074 appears to exert different effects between haplotypes, for example, *AGAC* (29% frequency) is associated with significant risk (all minor regulatory alleles are associated with enhanced expression) while other *G* haplotypes show lesser ORs (e.g.*, AGAG* OR < 1) (Fig. 3). Taken together, these results demonstrate differences between the main effects of rs16969968 alone in comparison to the haplotypes based on four SNPs.

We next address genotype effects of rs16969968 in the context of diplotypes (Fig. 3). Taken alone, the OR for heterozygous rs16969968 *G/A* carriers is 1.22 (95% confidence intervals: 1.03–1.44), whereas it is 1.88 (1.43–2.49) for homozygous *AA*, suggesting a non-linear superadditive effect. Subjects homozygous for the rs16969968 *AA* genotype are assigned only one of two prevalent diplotypes (*GAGC-GAGC* (9%) and *GAGC-GAGG* (3%)) and display the highest odds ratios (>two fold), when compared to the *AGAC-GGGG* diplotype assigned an OR of 1.0 (Fig. 3). The minor rs4887074 *G* allele does not appear to alter risk, consistent with the haplotype analysis. Subjects homozygous for the major rs16969968 *GG* alleles are carriers of seven assignable diplotypes (blue circles in Fig. 3, bottom), displaying ORs ranging from 0.82 to 1.63 (blue circles in Fig. 3, bottom). The major *C* allele of rs4887074 is distributed throughout the spectrum of diplotype OR values and is associated with significant risk in the diplotype *AGAC-AGAC* (OR = 1.63), a diplotype associated with high relative expression of all three genes (Table 3). Similarly, the other haplotype homozygous for both rs16969968 *G* and rs4887074 *C* (*AGAC-AGGC*) also is significant, but at a lower OR = 1.18 (Table 4).

**Table 4:** Comparison of odd ratio means among the diplotypes that share the same allele of rs16969968 (A) *GG* and (B) *AG*, along with predicted effects on *CHRNA5, CHRNA3, CHRNB4* mRNA expression

Diplotype	Percent	OR	CHRNA5	CHRNA3	CHRNB4
A. Diplotype with rs16969968 = GG					
*AGGC-GGGG*	2	0.82	medium	low	medium
*AGAC-GGGG*	8	1.00	medium	medium	medium
*GGGC-GGGG*	2	1.04	low	low	medium
*GGGG-GGGG*	2	1.07	low	low	low
*AGAC-GGGC*	4	1.08	medium	medium	high
AGAC-AGGC^*^	5	1.18	high	medium	high
AGAC-AGAC^*#^	8	1.63	high	high	high
B. Diplotype with rs16969968 = *AG*					
*AGAC-GAGG*	5	0.86	medium	medium	medium
*GAGC-GGGC*	4	1.05	low	low	high
*GAGC-GGGG*	8	1.32	low	low	medium
*AGAC-GAGC*^+^	18	1.62	medium	medium	high
*AGGG-GAGC*^+^	3	1.62	medium	high	medium
*AGGC-GAGC*^+^	5	1.68	medium	low	high

Expression level was defined as follows; homozygous minor alleles of the enhancer (rs880395 and rs1948) (or repressor, rs4887074) of each gene = ‘high’ (or ‘low’ with repressor), heterozygous genotype = ‘medium’; homozygous minor of enhancer (or repressor) = ‘low’ expression (or ‘high’ when a repressor)

^*^p < .05, Tukey test, pairwise comparison with *AGGC-GGGG*

^#^*p* < .05, Tukey test, pairwise comparison with *AGAC-GGGG*

^+^*p* < .05, Tukey test, pairwise comparison with *AGAC-GAGG*

Subjects heterozygous for rs16969968 *AG* alleles cover a diplotype range of ORs from 0.86 to 1.68, with two diplotypes conveying risk (but only the most frequent being significant: *AGAC-GAGC* (18%)), and three failing to show nominal risk. Note that the minor rs16969968 *A* allele is mostly on the same haplotype as the major rs4887074 *C* allele. These results suggest that rs4887074 does influence risk in our analysis, but in an allele-specific fashion: no effect is detectable if on the same haplotype with the rs16969968 *A* alelle, but the *C* allele appears to confer additional risk when together with the main rs16969968 *G* allele. This will be tested further below as a vQTL to detect allele selective interactions.

### Assessment of SNPs in LD bin 4, including rs6495308

Liu et al. have shown in their GWAS meta-analysis that rs6495308 is strongly associated with smoking quantity (6 × 10^− 44^; MAF = 0.24; protective effect), an association that remains significant after conditioning on rs16969968 (1.5 × 10^− 5^; MAF = 0.37) [10], even though the LD rs6495308/ rs16969968 has a D’ = 1 (R^2^ = 0.18 owing to different MAF; Table 2), with the minor alleles on opposite strands. To resolve this paradox, we built a 5-SNP haplotype from a European population of the 1000 Genomes project (Additional file 1: Table S5). The minor *C* allele of rs6495308 is almost exclusively present on two haplotypes also carrying the main *G* allele of rs169699698 (*GGGG* and *GGGC*), both conveying the least risk (OR~ 1 in Fig. 3) for nicotine dependence. Hence, rs6495308 is excluded from other haplotypes carrying the main *G* allele of rs16969968 that tend to convey risk, specifically the *AGAC* haplotype (Fig. 3). We propose that these hidden haplotype relationships drive the *p* values of the GWAS single SNP meta-analysis, including conditioning of one SNP on the other. These results further suggest that rs6495308 is not directly a marker of a single functional variant but rather tags specific haplotypes that are protective relative to other haplotypes in the gene cluster. However, we cannot exclude the possibility that rs6495308, or any other SNP associated with any of the 4-SNP haplotypes, does have functional significance that could be revealed by studying other ethnic groups with distinct LD patterns.

### Use of machine learning to integrate the effect of multiple SNPs

Using a cross-validated machine learning (penalized regression) approach, we determine how much these candidate variants increase the out-of-sample classification accuracy of nicotine dependence and non-dependence. Comparing AUC values of three different models (1 SNP: rs16969968 vs. 3 SNPs: rs880395-rs16969968-rs1948 vs. 4 SNPs: rs880395-rs16969968-rs1948-rs4887074), we find that the three models led to similar AUC values (mean AUC = 0.67 in all three models; see Additional file 1: Figure S2A). None of the models reveal a strong genetic influence beyond a model incorporating covariates (gender and age).

### Heteroscedasticity in interaction models (variance QTL analysis)

**Fig. 4:**
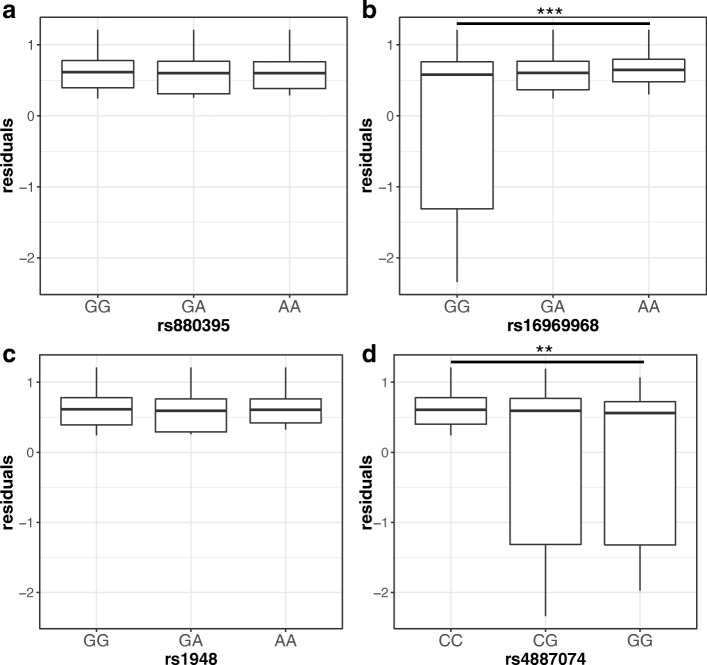
Distribution of the residuals probabilities of nicotine dependence score (FTND 0 or 4~ 10) associated with each of rs880395 (**a**), rs16969968 (**b**), rs1948 (**c**) and rs4887074 (**d**) alleles. To test heteroskedasticity of the residuals, the Breusch-Pagan test was performed. Among four SNPs, rs16969968 and rs4887074 are significantly associated with the variability of the residuals (**p < .01, ***p < .001)

To test the hypothesis that each of the four SNPs is subject to allele-specific interactions with other factors, we tested heteroscedasticity using the Breusch-Pagan test. Presented in Fig 4, rs880395 and rs1948 are not associated with residual probability of nicotine dependence, and the underlying models are homoscedastic (Fig. 4a and c; *p* = 0.92 and 0.70 respectively) – that is the variance does not differ between genotype. In contrast, the major *GG* genotype of rs16969968 robustly increases the residual probability of nicotine dependence compared to a heteroscedastic logistic model (Fig. 4b; *p* = 0.0005), suggesting an interaction with other variants or factors. In addition, the minor allele of rs4887074 in both *CG* and *GG* genotypes conveys significantly increased variance in the heteroscedastic model of nicotine dependence (Fig. 4d; *p* = 0.003). The minor *G* allele is associated with lower expression of *CHRNB4* and appears to be protective (Table 4). As the minor allele of rs4887074 is primarily on the same strand as the major allele of rs16969968, these data suggest a non-linear interaction between these two variants, while not excluding other interacting factors external to the gene cluster.

## Discussion

This study shows that rs4887074 *C > G*, tagging an LD bin associated with *CHRNB4* expression is significantly associated with nicotine dependence, the minor *G* allele conveying a protective effect in GWAS datasets (COGEND, UW-TTURC, SAGE) (Table 3). We then integrate the effect of rs4887074 with a previous analysis of 3-SNP haplotypes that modulate the functions of *CHRNA5* and *CHRNA3*, namely rs880395/ rs16969968/ rs1948) [11]. Owing to extended LD structures, haplotypes and diplotypes can be assigned with high confidence to a substantial portion of individual subjects for the 3-SNP haplotypes [11] and the 4-SNP haplotypes rs880395/ rs16969968/ rs1948/ rs4887074. The results further differentiate the modifying effects in haplotypes observed with the 3-SNP model [11] in the 4-SNP model. Subjects carrying the minor rs16969968 *A* allele resides on only one of two haplotypes that convey the strongest risk. On the other hand, 4-SNP diplotypes homozygous for the major rs16969968 *G/G* allele (previously thought to be protective) range from protective to conveying risk, which is significant for *AGAC-AGAC*. In this risk diplotype, the three regulatory tagging SNPs convey high expression for each *CHRNA3*, *CHRNA5,* and *CHRNB4*.

Estimated diplotypes reveal a gradation of effects ranging from ORs < 1 to > 2. Increased risk appears to be associated with enhanced expression of *CHRNA3*, *CHRNA5,* and *CHRNB4*; however, in the context of the rs16969968 *A* allele, the two risk haplotypes convey mostly low expression (*GAGC-GAGC* and *GAGC-GAGG*). We had previously suggested that rs880395 (enhancing *CHRNA5* expression) might convey a protective effect in the presence of the rs16969968 *A* allele by diluting the effect of the nsSNP, but the 4-SNP haplotypes analysis here indicates that the rs880395 effect is tends to a risk factor.

### Relationship of the 4-SNP haplotypes with previous haplotype-tagging SNPs involved in nicotine dependence

Numerous GWAS have identified the *CHRNA5/CHRNA3/CHRNB4* gene cluster as the region harboring the strongest association with nicotine dependence. In a large-scale meta-analysis of GWAS, Liu et al. [10] have identified three highly significant haplotype tagging SNPs, namely rs16969968 (haplotype A), rs588765 (haplotype B), and rs6495308 (haplotype C, protective), of which haplotype A and C yielded the strongest signals. Our LD analysis identifies 4 LD bins, with bin 1 identical to haplotype A (rs16969968), bin 2 with haplotype B (rs880395 and rs1948, which both appear to have independent effects) [11], bin 3 with rs4887074 that is not represented by top GWAS tagging SNPs in the three haplotypes of Liu et al. [10], and bin 4 from which we had not selected a lead tagging SNP, where however the strong tagging SNP rs6495308 is located (haplotype C). To determine whether rs6495308 represents a region with yet another regulatory variant or is tagging select 4-SNP haplotypes, we estimated the distribution of rs6495308 among the assignable 4-SNP haplotypes (rs880395-rs16969968-rs1948-rs4887074) (Additional file 1: Table S5). The results show that the minor *C* allele of rs6495308 is preferentially embedded in two haplotypes with the lowest risk profile (*GGGG* and *GGGC*). This finding indicates that the minor *C* allele of rs6495308 shows a protective effect resulting from its LD with these two protective 4-SNP haplotypes, a relationship that fails to be revealed from pairwise 2-SNP LD analysis alone. While rs6495308 also serves as an eQTL (GTEx) for *CHRNA3* in multiple tissues, with *p* = 0.0001 and NES = − 0.55 in nucleus accumbens, rs2869546 located next to rs6495308 is a stronger eQTL with *p* = 1.7e-13 and NES = − 0.72. On the other hand, the *CHRNA3* SNP rs1948 included in our 4-SNP haplotype has an eQTL with *p* = 6e-15 and NES = − 0.81. These data raise doubt whether rs6495308 represents a regulatory variant but rather fortuitously tags two protective haplotypes. However, additional regulatory variants could exist and show independent effects in non-European populations with distinct LD patterns.

### Interactions of four SNPs affecting nicotine dependence

We propose that the 4-SNP haplotype reflect combined additive effects, but also integrates epistatic non-linear interactions not captured by conventional methods. The four selected SNP in the *CHRNA5/A3/B4* gene locus can interact with each other in multiple ways. Because of the extensive LD structure, the presence of one allele affects the allele frequency of all other alleles in a given individual. For example, presence of the rs16969968 *A* allele excludes the rs880395 *A* allele in the same haplotype. To search for dynamic interactions between the 4 SNPs, a variance QTL analysis revealed that rs16969968 and rs4887074 appear to interact with each other (Fig. 4). Increased variance is observed only for the minor rs4887074 and the major rs16969968 alleles, consistent with the finding that rs4887074 is present only on rs16969968 *G* allele haplotypes with widely varying ORs (Fig. 3). We propose that rs4887074 has robust effects contingent on rs16969968 genotype.

### Odd ratios vs. AUC in machine learning

We here compare two different approaches - estimation of disease probability (odd ratios) and classification of subjects with their risk (AUC, machine learning) in the present study. Homozygous rs16969968 *AA* genotype and select diplotypes display robust association measured by ORs but are not strong classifiers. Considering the relationship between risk estimation (OR) and classification (sens; sensitivity, spec: specificity) with OR = sens/(1-sens)*spec/(1-spec), a SNP can indeed show a strong association (large odd ratios) but be a poor classifier [32, 33]. As nicotine dependence is a complex phenotype, future studies with machine learning algorithms addressing interactions between variants from multiple loci and with environmental factors are needed to improve model predictions.

### The relevance of distinct nicotine receptor configurations and genetic effects between brain regions

Composition of nicotinic receptors and tissue selective genetic effects of *CHRNA5/A3/B4* regulatory variants impinged on nicotine addiction in the reward circuit [34]. The medial habenula (MHb) is regarded as the key component regulating nicotine dependence via the α5, α3, and β4 subunits [35–37]. Elevated β4 expression in MHb decreases nicotine consumption by regulating nicotine aversion [38], while elevated expression in other regions may be risk factors. The genetic influence of rs4887074 or a regulatory variant in high LD needs to be studied in relevant brain regions before the clinical association observed here can be understood in functional terms. An example of region-selective genetic effects, rs880395 is associated with both *CHRNA3* and *CHRNA5* expression in most tissues, whereas in nucleus accumbens and putamen, rs1948 is the strongest eQTL. Thereby, regional differences in receptor subunit composition and genetic effects can lead to opposing effects and variable associations with distinct phenotypes.

## Conclusions

We have assessed the genetic influence of four variants in the *CHRNA5/A3/B4* gene cluster, employing a 4-SNP haplotype/diplotype analysis, adding the lead regulatory *CHRNB4* SNP rs4887074 to the 3-SNP haplotypes characterized previously [11]. Our approach further refines the cluster’s genetic influence on nicotine dependence, an important step if one considers evaluating the interaction between this gene cluster with other gene loci implicated in nicotine dependence. When attempting to detect dynamic non-linear interactions between loci, even small misrepresentations of the true genetic impact of each can lead to failure of detecting such dynamic effects [12, 17]. More broadly, our approach is generally applicable for studying integrated gene clusters and their genetic influence on any trait.

## Additional file

Additional file 1: **Table S1.** Haplotype of rs6495308 with lead SNPs of bin 1 (rs16969968, *G > A*) or bin 2 (rs1948, *G > A*) in European population (1000 Genomes project). **Table S2.** Effect size and *p* value for single SNPs and for rs16969968 in combination with the three regulatory lead SNPs in nicotine dependence. **Table S3.** Diplotypes and their frequency generated from (SAGE and Smoking Cessation dataset). **Table S4.** The ORs and confidence intervals of nicotine dependence with rs16969968 allele alone (*G > A*) and haplotype and diplotype comprised of rs880395 (*G > A*), rs16969968 (*G > A*), rs1948 (*G > A*) and rs4887074 (*C > G*). **Table S5.** 5-SNP (rs880395_rs16969968_1948_rs4887074_rs6495308) haplotypes distribution of European population from 1000 Genomes Project. **Figure S1.** Posterior probabilities of haplotypes assigned to each subject of GWAS dataset, estimated with the Expectation Maximization (EM) algorithm. **Figure S2.** Machine learning analysis comparing the prediction rate (AUC) of only covariates (sex and age) vs. 1 SNP vs. 3 SNPs vs. 4 SNPs models associated with nicotine dependence. (DOCX 257 kb)

## Abbreviations

AUC: Area under the curve
*CHRNA*: Cholinergic Receptor Nicotinic Alpha
*CHRNB*: Cholinergic Receptor Nicotinic Beta
COGEND: Collaborative Genetic Study of Nicotine Dependence
GWAS: Genome wide association study
LD: Linkage disequilibrium
MAF: Minor allele frequency
OR: Odd ratios
QTL: Quantitative trait loci
SAGE: Study of Addiction: Genetics and Environment
UW-TTURC: University of Wisconsin Transdisciplinary Tobacco Use Research Center
