# Supplementary material for: Combined genetic influence of the nicotinic receptor gene cluster CHRNA5/A3/B4 on nicotine dependence
Source: BMC Genomics. 2018 Nov 20;19:826. doi: 10.1186/s12864-018-5219-3 (PMC6245894; doi:10.1186/s12864-018-5219-3)
Supplement: Supplementary file 1 — Table S1. Haplotype of rs6495308 with lead SNPs of bin 1 (rs16969968, G > A) or bin 2 (rs1948, G > A) in European population (1000 Genomes project). Table S2. Effect size and p value for single SNPs and for rs16969968 in combination with the three regulatory lead SNPs in nicotine dependence. Table S3. Diplotypes and their frequency generated from (SAGE and Smoking Cessation dataset). Table S4. The ORs and confidence intervals of nicotine dependence with rs16969968 allele alone (G > A) and haplotype and diplotype comprised of rs880395 (G > A), rs16969968 (G > A), rs1948 (G > A) and rs4887074 (C > G). Table S5. 5-SNP (rs880395_rs16969968_1948_rs4887074_rs6495308) haplotypes distribution of European population from 1000 Genomes Project. Figure S1. Posterior probabilities of haplotypes assigned to each subject of GWAS dataset, estimated with the Expectation Maximization (EM) algorithm. Figure S2. Machine learning analysis comparing the prediction rate (AUC) of only covariates (sex and age) vs. 1 SNP vs. 3 SNPs vs. 4 SNPs models associated with nicotine dependence. (DOCX 257 kb) [file 12864_2018_5219_MOESM1_ESM.docx]

Table S1. Haplotype of rs6495308 with lead SNPs of bin 1 (rs16969968, *G>A*) or bin 2 (rs1948, *G>A*) in European population (1000 Genomes project). Numbers represent the frequency of each haplotype (%). The minor allele of rs6495308 resides mostly on the main *G* allele of rs16969968 and rs1948. These results suggest that rs6495308 (bin 4) appears as an eQTL because of opposite LD with rs1948.

| rs6495308 (*T*>*C*)_rs16969968(*G*>*A*) | | rs6495308 (*T*>*C*)_rs1948 (*G*>*A*) | |
| --- | --- | --- | --- |
| *T_G* | 40% | *T_G* | 48% |
| *T_A* | 38% | *T_A* | 31% |
| *C_G* | 21% | *C_G* | 20% |
| *C_A* | 0% | *C_A* | 1% |

Table S2. Effect size and p value for single SNPs and for rs16969968 in combination with the three regulatory lead SNPs in nicotine dependence (General Linear Model).

|  | Single SNP^a^  *p value (effect size)* | with rs16969968^b^  *p value (effect size)* |
| --- | --- | --- |
| rs880395 | 0.11 (-0.09) | 0.17 (0.10) |
| rs16969968 | 7.8e-06 (0.27) | - |
| rs1948 | 0.76 (-0.02) | 0.03 (0.15) |
| rs4887074 | 1.2e-03 (-0.21) | 0.013 (-0.17) |

^a^Logistic regression model per SNP : case ~ SNP + sex + age

^b^Logistic regression model with rs16969968 : case ~ SNP + rs16969968 + sex + age

Table S3. Diplotypes and their frequency generated from (SAGE and Smoking Cessation dataset) (n=3661)

| Diplotype | Number | Frequeny (%) |
| --- | --- | --- |
| *AGAC-GAGC* | 653 | 17.8 |
| *GAGC-GAGC* | 333 | 9.1 |
| *AGAC-AGAC* | 307 | 8.4 |
| *AGAC-GGGG* | 295 | 8.1 |
| *GAGC-GGGG* | 292 | 8.0 |
| *AGAC-AGGC* | 174 | 4.8 |
| *AGGC-GAGC* | 170 | 4.6 |
| *AGAC-GAGG* | 165 | 4.5 |
| *GAGC-GGGC* | 155 | 4.2 |
| *AGAC-GGGC* | 151 | 4.1 |
| *GAGC-GAGG* | 120 | 3.3 |
| *AGGG-GAGC* | 93 | 2.5 |
| *AGGC-GGGG* | 84 | 2.3 |
| *GGGC-GGGG* | 79 | 2.2 |
| *GGGG-GGGG* | 71 | 1.9 |
| *AGAC-AGGG* | 54 | 1.5 |
| *AGGC-GGGC* | 50 | 1.4 |
| *AGAC-AGAG* | 45 | 1.2 |
| *GAGC-GGAC* | 43 | 1.2 |
| *AGAC-GGAC* | 40 | 1.1 |
| *GAGG-GGGG* | 32 | 0.9 |
| *AGGC-AGGC* | 28 | 0.8 |
| *AGGG-GGGG* | 27 | 0.7 |
| *GGAC-GGGG* | 21 | 0.6 |
| *AGGC-AGGG* | 19 | 0.5 |
| *AGAC-GGAG* | 19 | 0.5 |
| *GAGC-GGAG* | 18 | 0.5 |
| *AGAG-GGGG* | 17 | 0.5 |
| *GGGC-GGGC* | 13 | 0.4 |
| *AGAG-GAGG* | 12 | 0.3 |
| *GGAC-GGGC* | 11 | 0.3 |
| *AGGG-GAGG* | 11 | 0.3 |
| *AAGC-GAGC* | 10 | 0.3 |
| *GAGG-GAGG* | 9 | 0.2 |
| *AAGC-AGAC* | 9 | 0.2 |
| *AGAC-GAAC* | 5 | 0.1 |
| *AAGC-AGGC* | 5 | 0.1 |
| *AGGG-AGGG* | 4 | 0.1 |
| *AGAG-AGGG* | 4 | 0.1 |
| *GGAG-GGGG* | 3 | 0.1 |
| *GAGG-GGAG* | 2 | 0.1 |
| *GAAC-GGAC* | 2 | 0.1 |
| *AGAG-AGAG* | 2 | 0.1 |
| *AAGC-AGAG* | 2 | 0.1 |
| *GGAG-GGAG* | 1 | 0.0 |
| *GAAC-GAGC* | 1 | 0.0 |

**Table S4. The ORs and confidence intervals of nicotine dependence with rs16969968 allele alone (*G>A*) and haplotype and diplotype comprised of rs880395 (*G>A*), rs16969968 (*G>A*), rs1948 (*G>A*) and rs4887074 (*C>G*)**

| haplotype | frequency(%) | OR | lower | upper |
| --- | --- | --- | --- | --- |
| *G* | 64 | 1 | 1 | 1 |
| *A* | 36 | 1.3 | 1.18 | 1.48 |
| *AGAG* | 2 | 0.78 | 0.59 | 1.02 |
| *GGGG* | 13 | 1 | 1 | 1 |
| *GGGC* | 7 | 1.02 | 0.88 | 1.17 |
| *AGGC* | 8 | 1.07 | 0.94 | 1.22 |
| *AGGG* | 3 | 1.18 | 0.94 | 1.48 |
| *AGAC* | 29 | 1.21 | 1.1 | 1.33 |
| *GAGG* | 5 | 1.32 | 1.09 | 1.59 |
| *GAGC* | 30 | 1.48 | 1.34 | 1.62 |
| *G-G* | 41 | 1 | 1 | 1 |
| *G-A* | 46 | 1.22 | 1.03 | 1.44 |
| *A-A* | 13 | 1.88 | 1.43 | 2.49 |
| *AGGC-GGGG* | 2 | 0.82 | 0.62 | 1.08 |
| *AGAC-GAGG* | 5 | 0.86 | 0.69 | 1.07 |
| *AGAC-GGGG* | 8 | 1.00 | 1.00 | 1.00 |
| *GGGC-GGGG* | 2 | 1.04 | 0.78 | 1.39 |
| *GAGC-GGGC* | 4 | 1.05 | 0.84 | 1.32 |
| *GGGG-GGGG* | 2 | 1.07 | 0.79 | 1.45 |
| *AGAC-GGGC* | 4 | 1.08 | 0.86 | 1.37 |
| *AGAC-AGGC* | 5 | 1.18 | 0.95 | 1.48 |
| *GAGC-GGGG* | 8 | 1.32 | 1.09 | 1.60 |
| *AGAC-GAGC* | 18 | 1.62 | 1.37 | 1.91 |
| *AGGG-GAGC* | 3 | 1.62 | 1.21 | 2.17 |
| *AGAC-AGAC* | 8 | 1.63 | 1.34 | 1.99 |
| *AGGC-GAGC* | 5 | 1.68 | 1.33 | 2.13 |
| *GAGC-GAGC* | 9 | 2.13 | 1.74 | 2.61 |
| *GAGC-GAGG* | 3 | 2.23 | 1.67 | 2.98 |

**Table S5. 5-SNP (rs880395_rs16969968_1948_rs4887074_rs6495308) haplotypes distribution of European population from 1000 Genomes Project**. The minor allele of rs6405308 (*C*, red) resides mostly on the major allele of rs16969968 (*G*, blue)

| haplotype | frequency (%) |
| --- | --- |
| *G_A_G_C_T* | 31.1 |
| *A_G_A_C_T* | 25.8 |
| *G_G_G_G_C* | 12.3 |
| *G_G_G_C_C* | 9.1 |
| *A_G_G_C_T* | 5.6 |
| *G_A_G_G_T* | 5.1 |
| *A_G_A_G_T* | 2.7 |
| *G_G_A_C_T* | 1.7 |
| *A_G_G_G_C* | 1.2 |
| *A_G_G_G_T* | 1.1 |

Fig S1. Posterior probabilities of haplotypes assigned to each subject of GWAS dataset, estimated with the Expectation Maximization (EM) algorithm. Nearly all haplotypes had a probability of >0.6, and >50% had a probability of 99% or higher.

**Fig S2.** **Machine learning analysis comparing the prediction rate (AUC) of only covariates (sex and age) vs. 1 SNP vs. 3 SNPs vs. 4 SNPs models associated with nicotine dependence.** AUC of covariates (sex + age) and covariate with 1 SNP (rs16969968), covariate with 3 SNPs (rs880395/rs16969968/rs1948) vs. covariate with 4 SNPs (rs880395/rs16969968/rs1948/rs4887074) in the test set. The AUC is only marginally improved over that achieved by demographic variables (sex and age) by any SNP model although 3 of 4 SNPs do show significant effects sizes individually.
